# Snf1/AMPK is involved in the mitotic spindle alignment in *Saccharomyces cerevisiae*

**DOI:** 10.1038/s41598-018-24252-y

**Published:** 2018-04-11

**Authors:** Farida Tripodi, Roberta Fraschini, Monica Zocchi, Veronica Reghellin, Paola Coccetti

**Affiliations:** 1 Department of Biotechnology and Biosciences, University of Milano-Bicocca, Milano, Italy; 2 SYSBIO, Centre of Systems Biology, Milan, Italy; 3 Present Address: Museo della Scienza e della Tecnologia Leonardo da Vinci, Milano, Italy; 4 Present Address: Eurofins BioPharma, Vimodrone, Italy

## Abstract

Before anaphase onset, budding yeast cells must align the mitotic spindle parallel to the mother-bud axis to ensure proper chromosome segregation. The protein kinase Snf1/AMPK is a highly conserved energy sensor, essential for adaptation to glucose limitation and in response to cellular stresses. However, recent findings indicate that it plays important functions also in non-limiting glucose conditions. Here we report a novel role of Snf1/AMPK in the progression through mitosis in glucose-repressing condition. We show that active Snf1 is localized to the bud neck from bud emergence to cytokinesis in a septin-dependent manner. In addition, loss of Snf1 induces a delay of the metaphase to anaphase transition that is due to a defect in the correct alignment of the mitotic spindle. In particular, genetic data indicate that Snf1 promotes spindle orientation acting in parallel with Dyn1 and in concert with Kar9. Altogether this study describes a new role for Snf1 in mitosis and connects cellular metabolism to mitosis progression.

## Introduction

During mitosis each cell must accurately segregate its chromosomes to ensure the faithful inheritance of genomic information. Improper segregation of DNA is the cause of many human diseases, most notably cancer and brain pathologies^1^. For cells that undergo asymmetric division, the correct alignment of the mitotic spindle along the polarity axis of the cell is crucial to ensure the fidelity of chromosome segregation. In *Saccharomyces cerevisiae*, the axis of cell polarity is established by the site of bud emergence^2^, named the bud neck, which determines the site of cytokinesis in the subsequent mitosis. Therefore, budding yeast cells must align the mitotic spindle parallel to the mother-bud axis to ensure that one chromosome set remains in the mother cell and the other passes through the bud neck into the daughter before cytokinesis^3^.

Two redundant pathways, that are regulated both in time and space, control the spindle orientation in budding yeast: the Kar9 pathway and the dynein pathway^4^. Inactivation of one of the two pathways has little effect on cell viability, but concomitant inactivation of both is lethal^5^. The early pathway of spindle orientation depends on Kar9, which is recruited to the daughter spindle pole body (SPB) by Bim1. Kar9-Bim1 complex translocates to the microtubule plus-ends where Kar9 binds Myo2, a class V myosin, resulting in polarized transport of the SPB and its microtubules along the actin cables into the bud, thereby positioning one SPB at the bud neck and leaving the other one in the mother cell^6^. The second pathway, or late pathway, acts during anaphase, when the daughter SPB is translocated through the bud neck to establish its final position along the cell polarity axis. This process requires the microtubule minus-end directed dynein–dynactin motor complex^7^.

If the mitotic spindle is misoriented, cells activate a surveillance mechanism, the spindle position checkpoint (SPOC), which delays mitotic exit until the spindle resumes the correct orientation. This is achieved through inhibition of the mitotic exit network (MEN)^8,9^, a signaling cascade that is controlled by the GTPase Tem1^10,11^ and leads the release of the Cdc14 phosphatase from the nucleolus to allow mitosis completion^12^. In response to spindle alignment defects, the SPOC holds MEN inactive by inhibiting the GTPase Tem1. Components of the SPOC are the Bfa1-Bub2 complex, the polo-like kinase Cdc5 and the kinase Kin4 together with the proteins involved in Kin4 regulation. Bfa1 and Bub2 form a bipartite GAP (GTPase activating protein) complex that accelerates GTP hydrolysis of Tem1, thus keeping Tem1 in the GDP-bound inactive form^13^ until the spindle has been properly oriented.

Protein kinase Snf1/AMPK is an energy sensor, conserved from yeast to humans^14^. In budding yeast, Snf1/AMPK is a complex made by the catalytic α subunit (Snf1), the γ subunit (Snf4) and one of the three alternative β subunits (Sip1, Sip2, Gal83), which determines the intracellular localization of the kinase^15,16^. Snf1/AMPK activation requires phosphorylation of the catalytic α subunit on Thr210 by one of three alternative kinases, Sak1, Tos3 or Elm1^17^. Snf1 activation also requires the association between α and γ subunits, which stabilizes the active conformation of the kinase^17^. In high glucose conditions, Snf1 is inactivated through dephosphorylation of Thr210 by the phosphatase Glc7/Reg1^18–20^. Upon activation, Snf1 phosphorylates a number of transcription factors, activating some and repressing others^16^. Specifically, active Snf1 causes the translocation to the cytoplasm of the inhibitor Mig1, responsible for the switch off of glucose repressed-genes^21,22^. Besides Mig1, Snf1 activates Cat8 and Sip4, which regulate the expression of gluconeogenic genes^23,24^, and the transcriptional activator Adr1^25^.

Although Snf1/AMPK function has been mostly studied in respiratory-dependent growth^26–28^, we have reported that Snf1 is required also in cells grown in 2% glucose, condition that supports maximal growth rate, showing its involvement in the regulation of the G1/S transition of the cell cycle^29,30^, as well as cellular metabolism^31^. Here we show that Snf1/AMPK has also a role in mitosis, as it contributes to the alignment of the spindle along the mother-bud axis, by acting in the Kar9-dependent pathway.

## Results

### Loss of Snf1 induces improper metaphase spindle alignment

**Figure 1:**
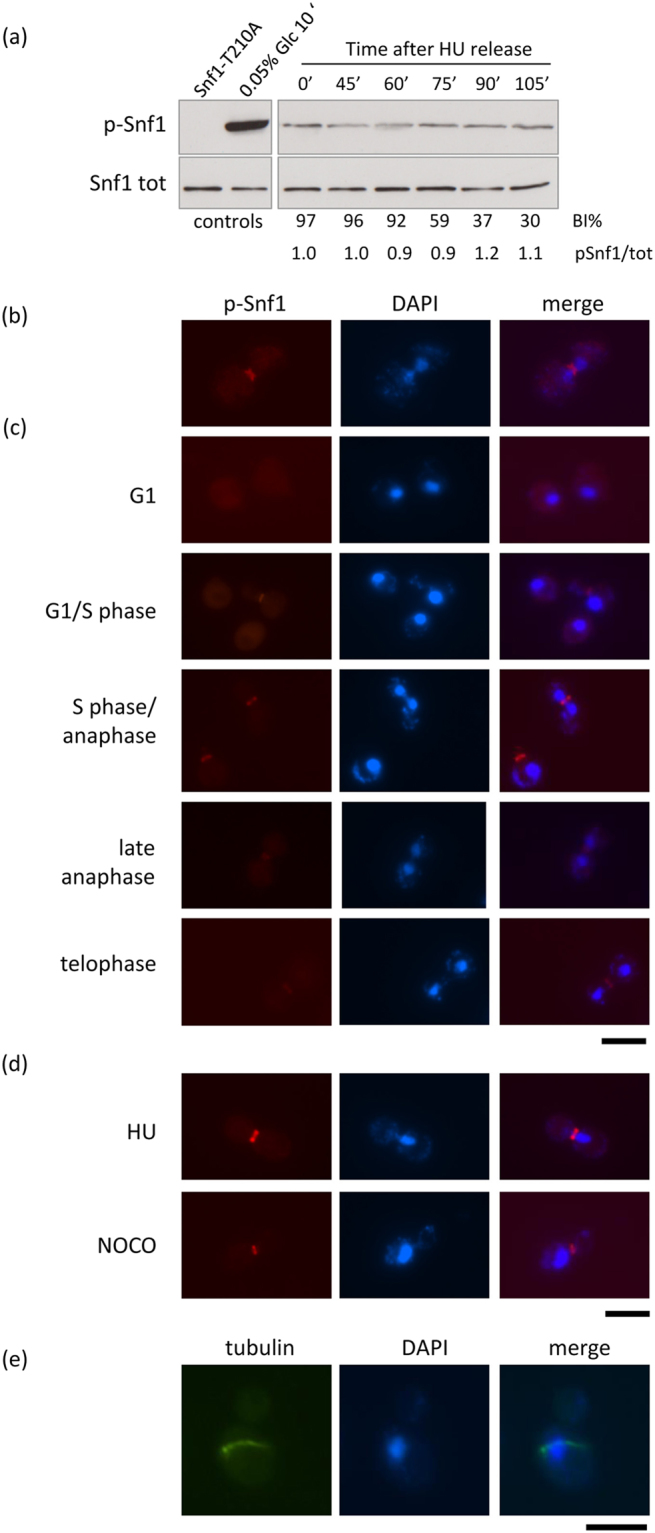
(**a**) A Snf1-HA expressing strain was grown in synthetic medium containing 2% glucose until exponential phase, then arrested and released from HU block as described in Materials and Methods. Samples were taken at different time points (0, 45, 60, 75, 90, 105 minutes) and analysed by western blot with anti-pT172-AMPK and anti-HA antibodies. Budding index (BI%) of cells at each time point and densitometric analysis of the pSnf1/tot ratio are shown. Cells expressing Snf1-T210A-HA and Snf1-HA cells shifted to 0.05% glucose for 10 min were used as negative and positive control, respectively. Controls were run together with samples and cropped as indicated by vertical lines. (**b**–**d**) Immunostaining for pT210-Snf1 using anti-pT172-AMPK antibodies and DAPI staining of DNA. bar: 5 µm. (**e**) Example of asynchronous *snf1Δ* cells with misaligned metaphase spindle. Spindle morphology (tubulin) and DAPI are shown. bar: 5 µm.

We reported that loss of *SNF1* induces a deep transcriptional change, with about 1000 deregulated genes^31^. Among these, Gene Ontology enrichment analysis highlighted several mitotic genes encoding both regulatory and structural proteins, most being down-regulated^31^. Thus, we wondered if this deregulation could indicate a requirement of protein kinase Snf1/AMPK in the mitotic phase of the cell cycle. We first investigated Snf1-T210 phosphorylation, which is indicative of a catalytically active kinase, during the cell cycle. Snf1-3HA expressing cells were synchronized in G1 phase by α-factor treatment, then released in medium containing hydroxyurea (HU) to block cells in S phase, and finally released in fresh medium containing α-factor. Samples were taken every 15 minutes from the release to focus on the mitotic phase. A slight and constant phosphorylation of Snf1-Thr210 was detectable, indicating that during mitotic progression Snf1 kinase is partially catalytically active (Fig. 1a, Supplementary Fig. S1a).

In glucose-repressed condition Snf1 is localized to the cytoplasm^32^, however specific localization of pT210-Snf1 during cell cycle phases has not been investigated yet. Thus, we performed indirect immunofluorescence analysis by using anti-pT172-AMPK antibodies to detect active Snf1 (p-Snf1) in asynchronous cells. Despite the low level of pT210-Snf1 detectable by western blot, specific immunofluorescence revealed a clear bud-neck localization of p-Snf1 in budded cells (Fig. 1b). We then analysed Snf1 localization in all cell cycle phases. Wild type cells were grown in synthetic media containing 2% glucose, synchronized in G1 phase by α-factor treatment and then released in fresh medium. Samples were taken at different times from the release, fixed and treated to analyse p-Snf1 localization by indirect immunofluorescence (Fig. 1c). p-Snf1 was not detectable in unbudded cells, while it was localized to the bud neck from bud emergence (late G1) to telophase in all budded cells, suggesting that active Snf1 in glucose growing cells is required for bud-neck related functions. We also induced mitotic arrest by treatment with HU, that causes dNTP depletion, or with nocodazole, that causes microtubules depolymerization, and interestingly we observed that both treatments caused p-Snf1 accumulation to the bud neck in all cells (Fig. 1d).

Since the bud neck plays a key role in mitotic spindle positioning, we analysed Snf1 role in this process. Asynchronous wild type cells and cells lacking Snf1/AMPK were fixed and mitotic spindles were visualized by indirect immunofluorescence. G1 monopolar spindle and anaphase spindle morphology of *snf1Δ* cells were indistinguishable from those of wt cells (data not shown), but interestingly about 30% of metaphase spindles were not properly oriented with respect to the cell polarity axis (Fig. 1e and Supplementary Fig. S1b), suggesting that Snf1/AMPK could be required for the correct spindle alignment. Consistently, cells expressing the catalytically inactive mutant Snf1-K84R showed the same defect of spindle alignment, while the constitutive active Snf1-G53R mutant was unaffected (Supplementary Fig. S1b), suggesting that Snf1 catalytic activity is required for proper spindle orientation.

### Active Snf1 co-localizes with septins and depends on proper septin structure

**Figure 2:**
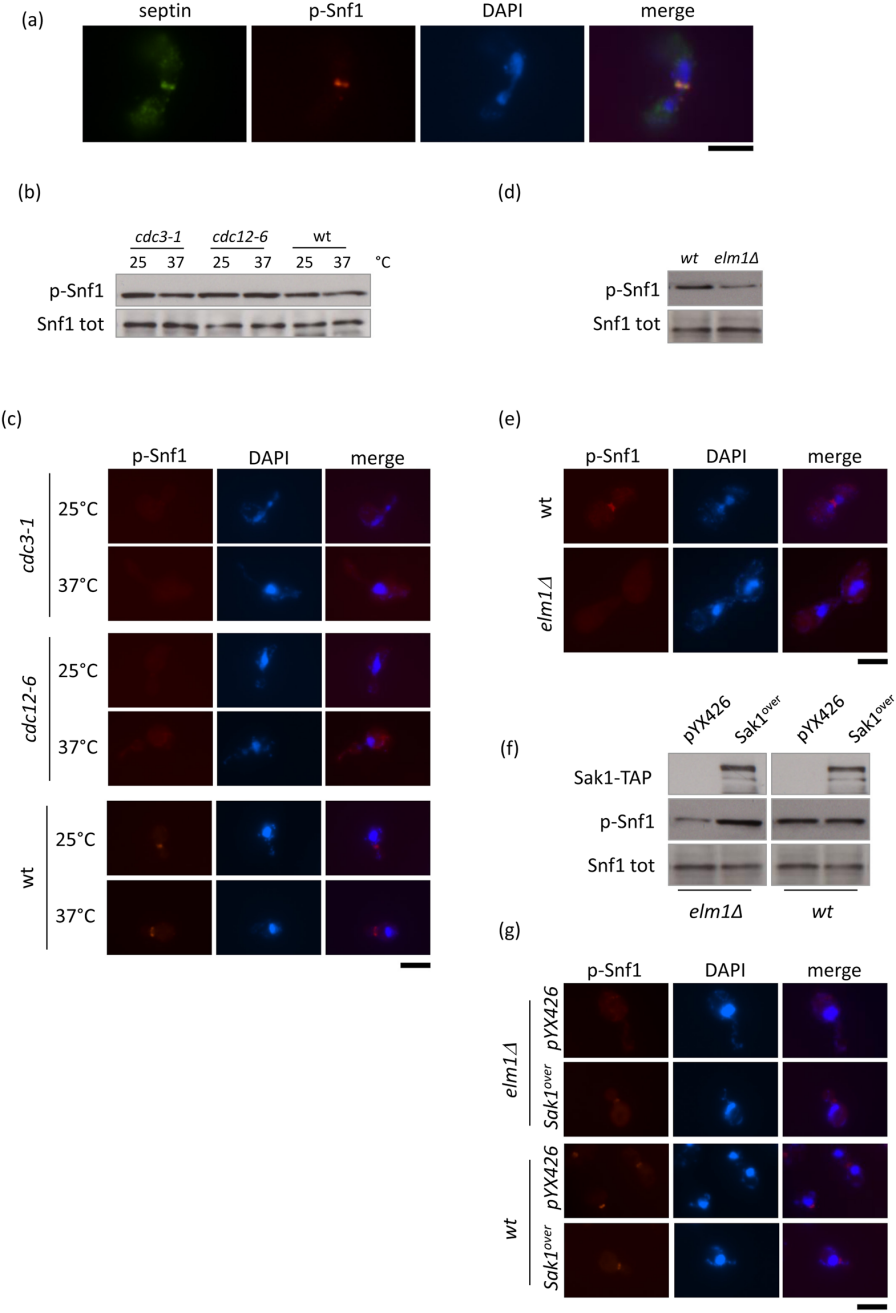
(**a**) Co-localization of pT210-Snf1 and Cdc3-HA by using anti-pT172-AMPK and anti-HA antibodies; DAPI was used for DNA. (b-c) *cdc3-1, cdc12-6* and wt cells were grown at 25 °C and then shifted for 2 hours at 37 °C. Sample were taken to evaluate **(b**) p-Snf1 and total Snf1 levels by western blot with anti-pT172-AMPK and anti-His antibodies and (**c**) p-Snf1 localization by immunostaining using anti-pT172-AMPK antibodies and DAPI staining of DNA. bar: 5 µm. (**d**,**e**) wt and *elm1Δ* cells were grown at 30 °C and analysed to evaluate (**d**) p-Snf1 and total Snf1 levels by western blot with anti-pT172-AMPK and anti-His antibodies and (**e**) p-Snf1 localization by immunostaining using anti-pT172-AMPK antibodies and DAPI staining of DNA. bar: 5 µm. (**f,g**) *elm1Δ[pYX426]*, *elm1Δ[pSAK1-TAP]*, *wt[pYX426]* and *wt[pSAK1-TAP]* cells were grown in synthetic medium containing 2% glucose until exponential phase. Samples were taken to assay (**f**) SAK1-TAP, p-Snf1 and total Snf1 levels by western blot with anti-TAP, anti-pT172-AMPK and anti-His antibodies (the gel was cropped as indicated by vertical lines) and (**g**) p-Snf1 localization by immunostaining using anti-pT172-AMPK antibodies and DAPI staining of DNA. bar: 5 µm.

Immunofluorescence experiments suggested that active Snf1 could localize to the septin ring, as in large budded cells p-Snf1 was positioned at the division site in an hourglass shape (Fig. 1b), typical of septin structure during mitosis. To test this hypothesis, we performed experiments of co-localization between p-Snf1 and Cdc3, a component of the septin ring^33^. Active Snf1 co-localized with Cdc3-HA in all budded cells (Fig. 2a). Then, we asked whether proper septin organization was necessary for p-Snf1 localization to the bud neck. Two different temperature-sensitive mutants (*cdc3-1* and *cdc12-6*), as well as the control wt strain, were grown at permissive temperature (25 °C) and then shifted for 2 hours at 37 °C to perturb septin structure. Although the global level of Snf1 phosphorylation was not altered in these cells (Fig. 2b), p-Snf1 localization to the bud neck was already impaired in septins mutants grown at 25 °C and almost absent after shift at 37 °C, while no defect was observed in the wt (Fig. 2c). These results suggest that the proper septin structure, which is still partially perturbed at permissive temperature^34,35^, is essential for Snf1 localization to the bud neck.

Snf1 phosphorylation depends on three redundant kinases (Elm1, Sak1, Tos3), one of which, Elm1, is also involved in the regulation of cellular morphogenesis, septin behavior and cytokinesis^36^. Therefore, we assayed whether p-Snf1 localization to the septin ring was dependent on Elm1. Total Snf1 phosphorylation was reduced in cells lacking Elm1 (Fig. 2d), in agreement with the reduced Snf1 activity previously reported^37^. Remarkably, p-Snf1 localization was not detectable in *elm1Δ* cells (Fig. 2e), which show long buds and altered septin organization^36^. Moreover, overexpression of Sak1^38^, which increased the total level of p-Snf1 (Fig. 2f) and mostly reverted the morphologic defect of the *elm1Δ* mutant, was also able to partially restore p-Snf1 localization to the bud neck (Fig. 2g). On the contrary, in the control wt strain, overexpression of Sak1 had no effect (Fig. 2f,g).

### Snf1/AMPK catalytic activity promotes progression through mitosis

**Figure 3:**
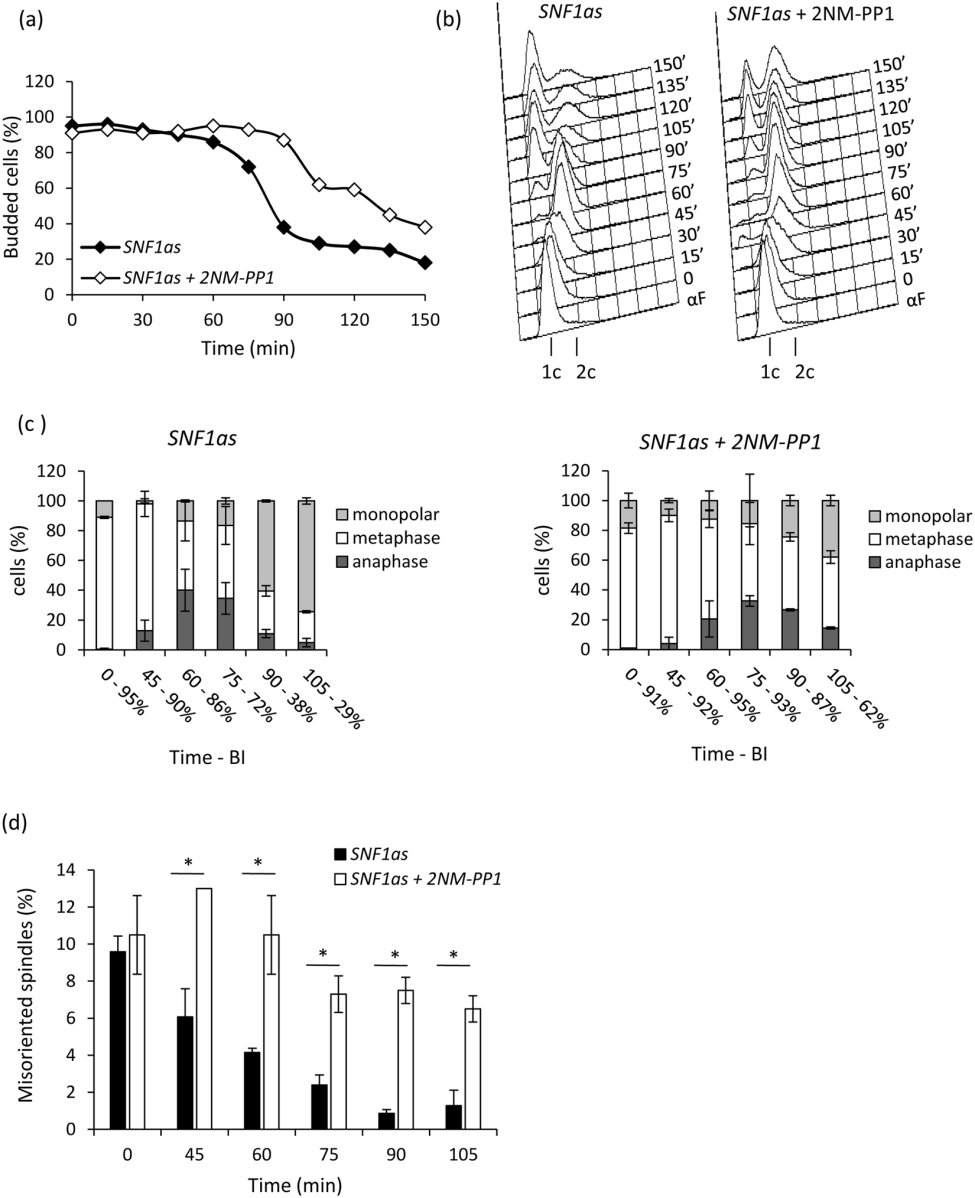
(**a**–**d**) *Snf1-as* cells were grown in synthetic medium containing 2% glucose, synchronized in S phase by HU as described in Materials and Methods and released in fresh medium containing α-factor and either 25 µM 2NM-PP1 or 0.1% DMSO (solvent control). Samples were taken at the indicated time points to assay (**a**) budding index, (**b**) DNA content by FACS analysis, (**c**,**d**) spindle morphology. *p < 0.05.

Although *snf1Δ* cells showed about 30% of misaligned metaphase spindles (Supplementary Fig. S1b), they could likely properly orient their spindle, since no defect in anaphase spindles was observed. Thus, we decided to analyse progression through mitosis in synchronous cells. To obtain a high degree of synchronization, cells were first blocked in G1 phase with α-factor and then released in medium containing hydroxyurea; when budding index was > 90%, they were washed and released in fresh prewarmed medium containing α-factor. To overcome problems connected with the altered progression through G1-phase of *snf1Δ* cells^29^, these experiments were performed in a strain expressing an analog-sensitive (as) allele of Snf1, whose activity was selectively inhibited only after release from HU block with 2-naphthylmethyl pyrazolopyrimidine 1 (2NM-PP1)^27^. After HU-release, cells with active Snf1 duplicated their DNA, progressed through mitosis and divided, as indicated by the drop of budding index and the appearance of cells with 1 C DNA content (Fig. 3a,b). Instead, in the presence of 2NM-PP1, cells could duplicate their DNA, but remained longer with large buds and cells with 1 C DNA content appeared with a delay of 30 minutes (Fig. 3a,b). Moreover, in keeping with budding index and DNA content, mitotic spindle elongation and disassembly occurred later than in control cells (Fig. 3c). A typical feature of HU arrested cells was the presence of misaligned metaphase spindles in about 10–15% of the population. Strikingly, while in non-treated cells the fraction of misaligned spindles rapidly decreased after the release from the block, it persisted much longer in cells in which Snf1 activity was inhibited (Fig. 3d), suggesting that Snf1 catalytic activity was required for the proper spindle orientation along the mother-bud axis. This orientation defect could account for the delay of spindle elongation and mitotic exit of cell lacking Snf1 activity.

We previously showed that the requirement of Snf1/AMPK for normal growth rate and proper G1/S transition depends on glucose concentration, being dispensable in 5% glucose-medium^29^. If this was the case also for mitosis, the mitotic defect above reported (Fig. 3) should be abolished in 5% glucose. Remarkably, the inhibition of Snf1 activity with 2NM-PP1 had no effect, either on budding index or on metaphase spindle re-orientation in HU-released cells in 5% glucose, (Supplementary Fig. S2a,b), further supporting the dispensability of Snf1 activity at this glucose concentration.

**Figure 4:**
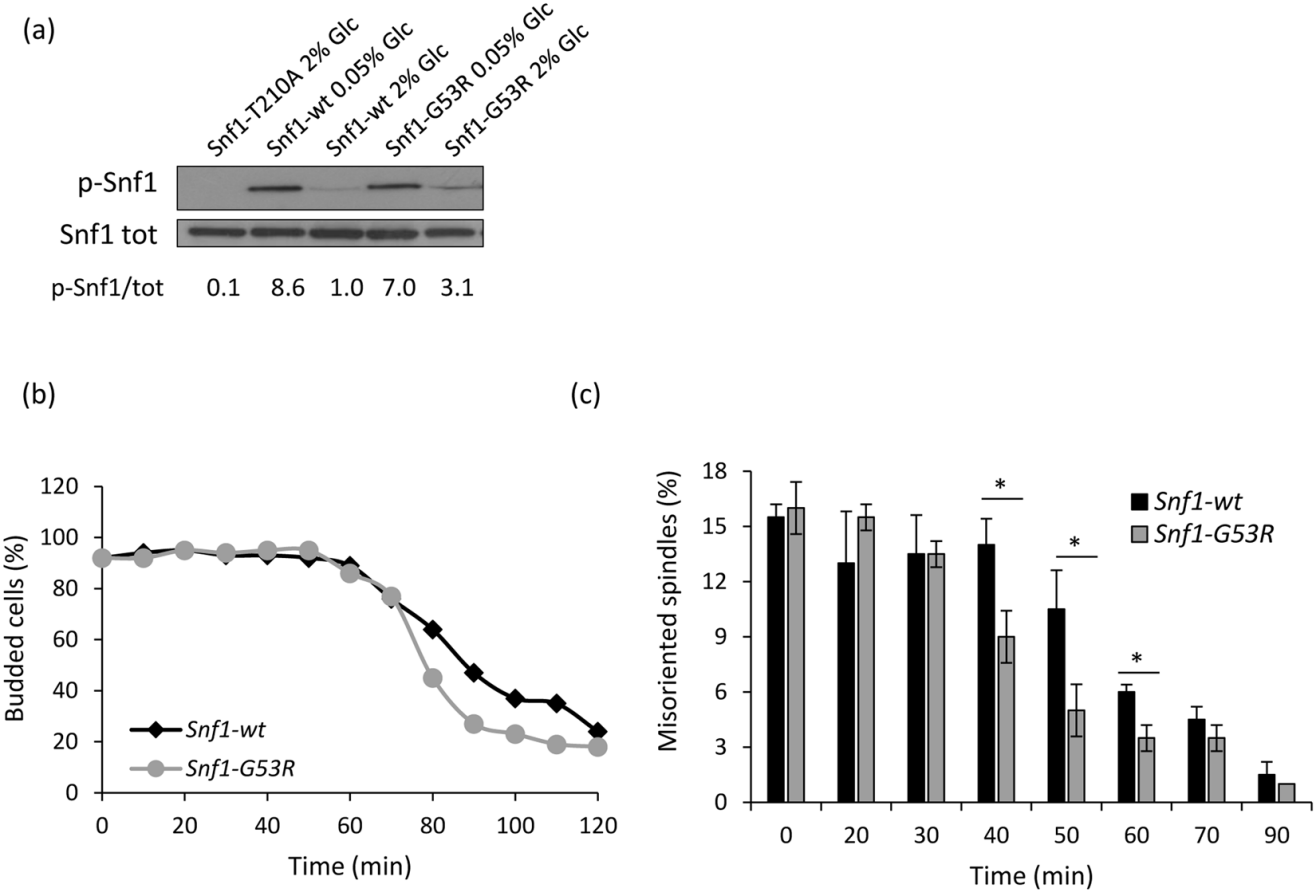
(**a**) *Snf1-T210A*, *Snf1-wt* and *Snf1-G53R* strains were grown in synthetic medium with 2% or 0.05% glucose, as indicated, and processed for western analysis with anti-pT172-AMPK or anti-HA antibodies. Densitometric analysis of the pSnf1/tot ratio is shown. (**b**,**c**) *Snf1-wt* and *Snf1-G53R* cells were grown in synthetic medium containing 2% glucose, synchronized in S phase by HU as described in Materials and Methods and released in fresh medium containing α-factor. Samples were taken at the indicated time points to assay (**b**) budding index, (**c**) spindle morphology. *p < 0.05.

Since inactivation of Snf1 caused a mitotic delay, probably due to an impaired ability in spindle orientation, we then investigated the effect of a constitutively active Snf1, by using the Snf1-G53R mutant, which presents a higher phosphorylation of Thr210 than the wild type under non-limiting glucose conditions^39^ (Fig. 4a). HU-released cells expressing Snf1-G53R mutant showed a slightly faster mitotic progression (Fig. 4b), and a faster spindle re-orientation kinetic if compared to the wild type (Fig. 4c), suggesting that a higher Snf1 activity could improve spindle orientation along the mother-bud axis.

### Cells lacking Snf1 activity correctly activate the SPOC

**Figure 5:**
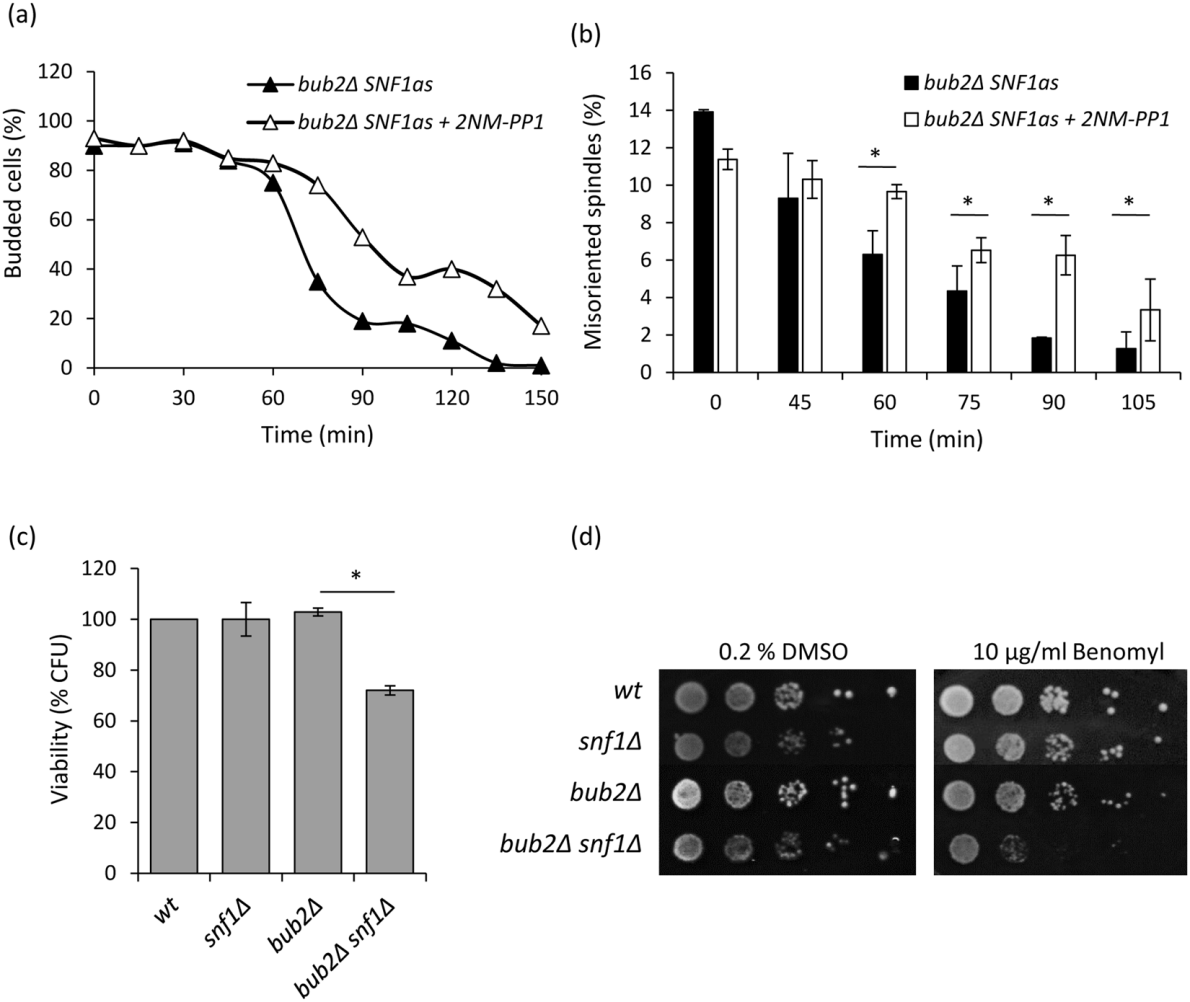
(**a**,**b**) *bub2Δ Snf1-as* cells were grown in synthetic medium containing 2% glucose, synchronized in S phase by HU as described in Materials and Methods and released in fresh medium containing α-factor and either 25 µM 2NM-PP1 or 0.1% DMSO (solvent control). Samples were taken at the indicated time points to assay (**a**) budding index, (**b**) spindle morphology. *p < 0.05. (**c**) Viability of wild type, *snf1Δ*, *bub2Δ* and *bub2Δsnf1Δ* cells. *p < 0.05. (**d**) Exponentially growing wild type, *snf1Δ*, *bub2Δ* and *bub2Δsnf1Δ* cells were serially diluted (1:10), and each dilution was spotted out onto YPD plates with 0.2% DMSO (solvent control) or 10 µg/ml benomyl. The plates were then incubated at 30 °C for 3 days.

The mitotic spindle orientation is monitored by the Spindle Position Checkpoint (SPOC), which delays mitotic exit by inhibiting the Mitotic Exit Network (MEN) until the proper orientation has been reached^3^. Data presented above suggest that *snf1Δ* cells are defective in spindle orientation, but can properly inhibit mitotic exit, thus presenting an active SPOC. Therefore, we decided to combine Snf1 inactivation with *BUB2* deletion, which abrogates the SPOC^8,9^. *bub2Δ SNF1as* cells released from HU arrest showed the phenotype typical of Snf1 inactivation, when 2NM-PP1 was added to the medium, with impaired ability of re-orient metaphase spindles (Fig. 5a,b). However, *bub2Δ snf1Δ* cells showed a 30% reduction of viability compared to parental *bub2Δ* and *snf1Δ* strains (Fig. 5c) and a higher sensitivity to the microtubule-depolymerizing agent benomyl (Fig. 5d), indicating that *snf1Δ* cells require an intact SPOC. In addition, lack of Bub2 did not impair p-Snf1 localization to the bud neck (Supplementary Fig. S3).

### SNF1 is epistatic with KAR9

**Figure 6:**
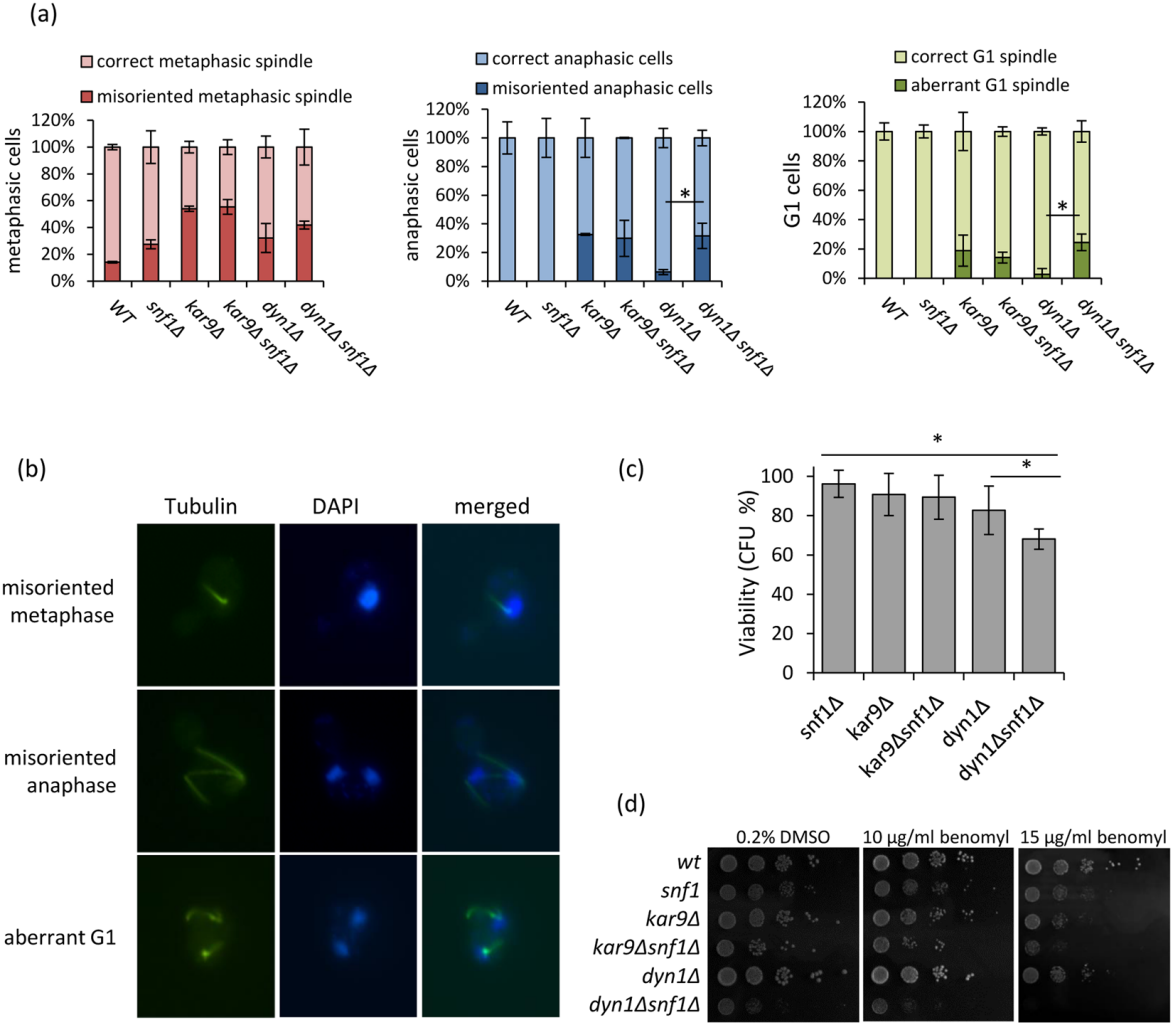
(**a**) Spindle morphology of wild type, *snf1Δ*, *kar9Δ* and kar9*Δsnf1Δ*, *dyn1Δ* and *dyn1Δsnf1Δ* in synthetic medium containing 2% glucose. *p < 0.05. (**b**) Examples of metaphasic and anaphasic cells with misoriented spindles and aberrant G1 cells (two nuclei) in *dyn1Δsnf1Δ* mutant. Spindle morphology was analysed with anti-tubulin antibody, DAPI was used to stain DNA. (**c**) Viability of *snf1Δ*, *kar9Δ* and kar9*Δsnf1Δ*, *dyn1Δ* and *dyn1Δsnf1Δ* cells. *p < 0.05. (**d**) Exponentially growing wt, *snf1Δ*, *kar9Δ* and kar9*Δsnf1Δ*, *dyn1Δ* and *dyn1Δsnf1Δ* cells were serially diluted (1:10), and each dilution was spotted out onto YPD plates with 0.2% DMSO (solvent control), 10 µg/ml benomyl or 15 µg/ml benomyl. The plates were then incubated at 30 °C for 3 days.

Two major pathways are responsible for the spindle alignment along the mother-bud axis: the Kar9 pathway and the Dyn1 pathway^9^. To discern in which pathway Snf1 could act, we combined *SNF1* deletion with deletion of *KAR9* or *DYN1* genes. Spindle analysis by immunofluorescence of cycling cells revealed that the double mutant *kar9Δ snf1Δ* had no additive effect on mitotic spindles if compared to the *kar9Δ* mutant (Fig. 6a). On the contrary, the double mutant *dyn1Δ snf1Δ* presented an aggravation of the phenotype, with defects not only in metaphase spindles, but also in other phases, with cells executing anaphase and dividing nuclei in the mother, or G1 cells without nucleus or presenting more than one nucleus (Fig. 6a,b). In keeping with these data, *kar9Δ snf1Δ* viability was not impaired if compared to the parental strains, while *dyn1Δ snf1Δ* exhibited an additive effect (Fig. 6c) and could not grow in the presence of the depolymerizing drug benomyl (Fig. 6d).

Altogether, these data suggest that Snf1 could act in the Kar9 pathway to promote metaphase spindle orientation.

### KAR9 deletion reduces Snf1 phosphorylation and localization to the bud neck

**Figure 7:**
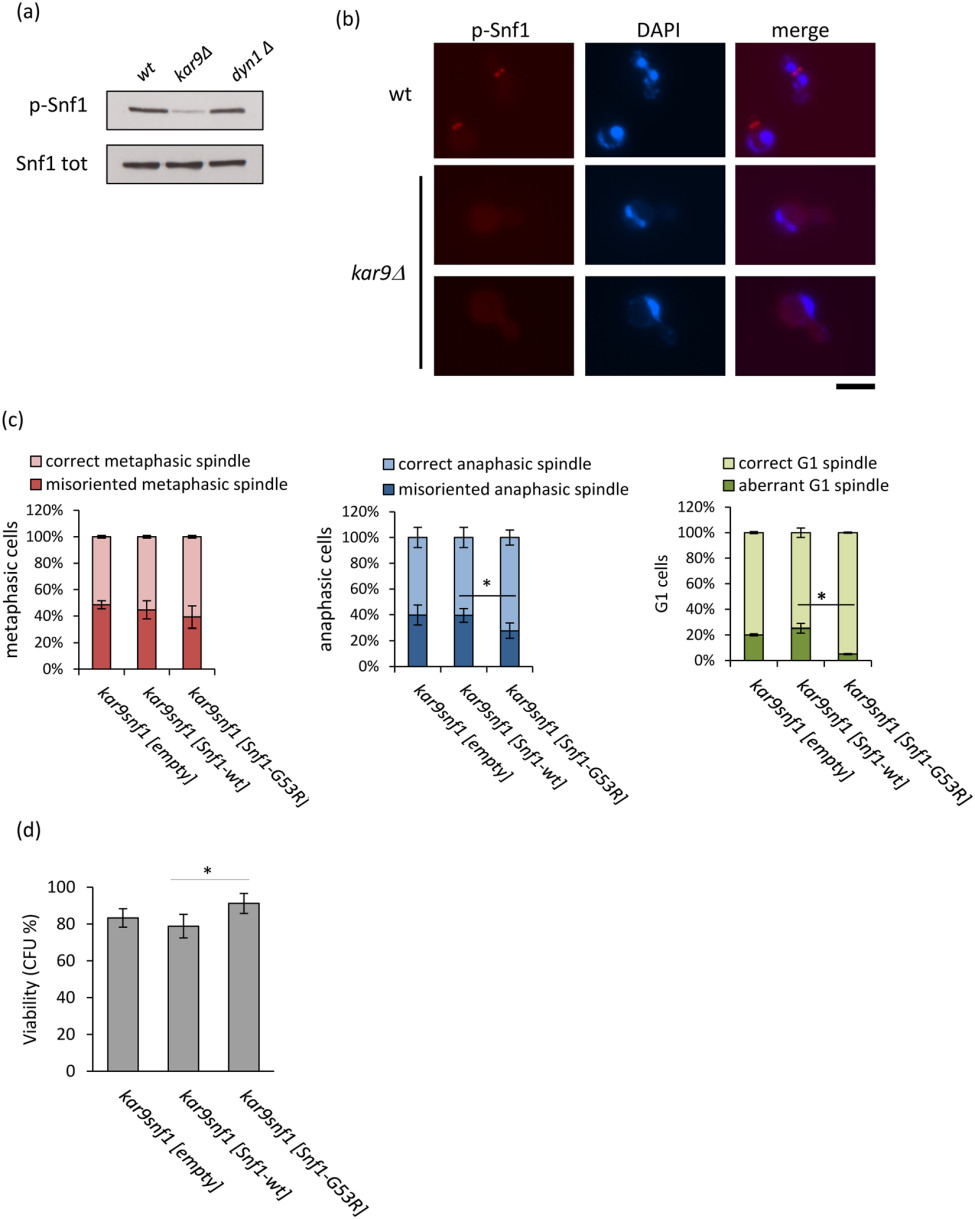
(**a**) *wild type*, *kar9Δ* and *dyn1Δ* strains expressing Snf1-3HA were grown in synthetic medium with 2% glucose and processed for western analysis with anti-pT172-AMPK or anti-HA antibodies. (**b**) Immunostaining for pT210-Snf1 using anti-pT172-AMPK antibodies and DAPI staining of DNA in wt and *kar9Δ* cells. bar: 5 µm. (**c**) Spindle morphology and (**d**) viability of *kar9Δsnf1Δ[empty], kar9Δsnf1Δ[Snf1-wt] and kar9Δsnf1Δ[Snf1-G53R]* in synthetic medium containing 2% glucose. *p < 0.05.

Then, we examined Snf1-T210 phosphorylation in strains lacking Kar9 or Dyn1. Surprisingly, Snf1 phosphorylation was strongly reduced in *kar9Δ* cells compared to wild type, while it was unaltered in *dyn1Δ* cells (Fig. 7a). Lack of Kar9 impaired p-Snf1 localization to the bud neck (Fig. 7b), although no defect in Elm1 localization was observed (Supplementary Fig. S4a). The overexpression of Sak1, which increased the total level of p-Snf1 (Supplementary Fig. S4b), was not able to revert the defective localization of p-Snf1 to the bud neck in *kar9Δ* cells (Supplementary Fig. S4c), suggesting that p-Snf1 localization depends on the integrity of the Kar9-pathway.

Nevertheless, we found that expression of Snf1-G53R in *kar9Δ* cells improved spindle morphology in asynchronous cells, with a reduction of misaligned anaphase spindles and an even more pronounced reduction of G1 cells without nucleus or presenting more than one nuclei (Fig. 7c). This improvement correlated with an increase of cell viability (Fig. 7d), indicating that a hyper-activation of Snf1 can ameliorate the phenotypes associated to *KAR9* deletion.

## Discussion

The protein kinase Snf1/AMPK is a highly conserved energy sensor that plays key roles during adaptation to glucose limitation and in response to cellular stresses. Our results uncover new functions of Snf1 in non-limiting glucose conditions. In the present study, we have shown that budding yeast Snf1 is active during mitosis and active Snf1 is localized at the division site from the time of bud emergence to cytokinesis (Fig. 1). We observed that both septins and their associated kinase Elm1 are required for proper Snf1 localization to the bud neck, indicating that the presence of an accurate scaffold is necessary for this process (Fig. 2). Since the bud neck structure and its associated proteins are essential for proper spindle positioning and orientation, we investigated Snf1 role in this process. Interestingly, we found that loss of Snf1 activity causes a defect in the correct alignment of the mitotic spindle, that in turn induces a delay of the metaphase-to-anaphase transition, thus indicating that Snf1 is required for proper spindle orientation. In addition, the concomitant loss of Snf1 and Dyn1 causes an additive defect in spindle positioning, while *SNF1* deletion does not exacerbate the *kar9Δ* mutant defects (Fig. 6). Moreover, a constitutive activation of Snf1 (using the Snf1-G53R isoform) ameliorates the phenotypes of a *kar9Δ* mutant and remarkably, both Snf1 activation and localization are impaired in a *kar9* background (Fig. 7). We can therefore hypothesize that Snf1 acts in parallel to Dyn1 and in concert with Kar9 to promote spindle positioning. It seems likely that Snf1 could phosphorylate one of the components of the Kar9-dependent pathway. Then, the identification of Snf1 targets in this pathway will be of interest to better understand its function during spindle orientation.

Interestingly, the human homolog of Kar9, APC (*adenomatous polyposis coli*), is involved in several processes, especially in the suppression of the Wnt signaling and in the regulation of spindle orientation and migration^40,41^. APC mutations are strongly associated to colorectal cancers (both sporadic and hereditary forms of the disease)^42^. Since we showed that constitutive activation of Snf1 improves spindle morphology and viability of *kar9Δ* cells, our study suggests investigating the effects of AMPK activation in APC-mutated cancer cells.

The role of Snf1/AMPK in the regulation of mitosis is not surprising, since it could be able to couple energy homeostasis and cell cycle. Indeed, we recently showed that Snf1 has a similar role also in G1 phase, where it contribute to the transcription of G1-genes^29,30^. The role of protein kinase AMPK in the regulation of mitosis has recently emerged also in higher eukaryotes, suggesting that it could act as a conserved energy checkpoint to coordinate cell growth and cell cycle progression. Vazquez-Martin and co-workers showed that AMPK is dynamically regulated during mitosis and transiently associates with mitotic structures, such as centrosomes, spindle poles, the central spindle midzone^43,44^. Moreover, mutation of the AMPK-activating kinase LKB1 impairs spindle orientation in cells and causes mis-localization of AMPK in mitotic cells^45^. Mitotic proteins are emerging as new substrates of AMPK^46^, although it was shown that some of these could be phosphorylated in an energy-independent manner, like MRLC (myosin II regulatory light chain), which regulates the transition from metaphase to anaphase^47^.

Therefore, the role of AMPK in the regulation of mitosis seems a quite conserved feature of eukaryotic cells, which deserves to be further investigated.

## Materials and Methods

### Yeast strains and growth conditions

*S. cerevisiae* strains used in this study are listed in Table S1. Cells were grown in synthetic media, prepared by assembling 2% glucose, 6.7 g/L yeast nitrogen base (Difco), 50 mg/L of required amino acids. For synchronization experiments, 2.5 μM α-factor was added to exponentially growing cells, then when budding index was below 5%, cells were released in medium containing 200 mM hydroxyurea; after 90–120 min, when budding index was >90%, cells were washed and released in fresh prewarmed medium containing 2.5 μM α-factor. For experiments with the *Snf1-as* strain, 25 μM 2NM-PP1 (from a 25 mM stock in 100% DMSO) was added to inhibit the activity of Snf1. In the control, the same concentration of solvent (0.1% DMSO) was added. Synthesis of compound 2NM-PP1 was carried out as previously described^30^.

### FACS analysis

A total of 1 × 10^7^ cells was collected by filtration for each sample, fixed in 70% ethanol and subsequently processed for FACS analysis. Cells were washed three times with PBS and incubated overnight in PBS plus 1 mg/ml RNAse (Roche). The day after, cells were washed once with PBS and resuspended in 46 µM propidium iodide (Sigma). Analysis was performed with a BD Biosciences FACSCalibur.

### Recombinant DNA techniques and genetic manipulations

DNA manipulations and yeast transformations were carried out according to standard techniques.

### Protein extraction and immunoblotting

Samples of cells were lysed using ice-cold lysis buffer (50 mM Tris-HCl pH 7.5, 150 mM NaCl, 0.1% NP-40, 10% glycerol) plus 1 mM PMSF (phenylmethanesulfonylfluoride), proteases inhibitor mix (Complete EDTA free Protease Inhibitor Cocktails Tablets, Roche) and phosphatase inhibitor mix (Cocktail 2, Sigma-Aldrich). Protein concentration was determined using the Bio-Rad protein assay. Western blot analysis was performed using anti-HA antibody (Roche), anti-phosphoT172-AMPK antibody (Cell Signalling), anti-His antibody (Sigma-Aldrich) or anti-TAP antibody (Thermo Fisher Scientific). Densitometric analysis was performed by using the ImageJ software (NIH, http://imagej.nih.gov/ij/).

### Fluorescence Microscopy

*In situ* immunofluorescence was performed on formaldehyde-fixed cells and carried out as previously described^48^. Nuclei were visualized by staining with DAPI 0.05 μg/ml. To study spindle morphology and positioning, α-tubulin immunostaining was performed with the YOL34 monoclonal antibody (1:100, Serotec) followed by indirect immunofluorescence using rhodamine-conjugated anti-rat antibody (1:500, Pierce Chemical Co). At least 150 cells were counted for each sample. Snf1 localization was observed in formaldehyde-fixed cells immunostained with anti-phospho-T172-AMPK antibody (1:50, Cell Signalling) and with TRITC-conjugated anti-rabbit antibody (1:100, Life Technologies). For the colocalization experiment, Cdc3-HA was detected with 16B12 monoclonal antibody (1:200, Babco) and with FITC-conjugated anti-mouse Alexa Fluor 488 antibody (1:500, Life Technologies). Digital images were taken with a Nikon DS-Qi MC camera mounted on a Nikon Eclipse 600 and controlled by the NIS elements imaging software (Nikon) with an oil 100 × 0.5–1.3 PlanFluor oil objective (Nikon).

### Viability assay

500 cells were plated on YPD medium and grown for 3 days. Viability was determined by colony-forming units (CFU), data are presented as mean ± S.D. of 3 independent experiments performed in triplicate.

### Statistical Analysis

Experiments were performed in triplicate and results are expressed as mean ± SD. Results were compared by using the two-sided Student’s t-test. Differences were considered statistically significant at p < 0.05.

### Data availability

All data generated or analysed during this study are included in this published article (and its Supplementary Information files).

## Electronic supplementary material

Supplementary Information
