# Supplementary material for: Snf1/AMPK is involved in the mitotic spindle alignment in Saccharomyces cerevisiae
Source: Sci Rep. 2018 Apr 11;8:5853. doi: 10.1038/s41598-018-24252-y (PMC5895576; doi:10.1038/s41598-018-24252-y)
Supplement: Supplementary file 1 — Supplementary Information [file 41598_2018_24252_MOESM1_ESM.pdf]

Supplementary Information

Snf1/AMPK is involved in the mitotic spindle alignment in Saccharomyces cerevisiae

Farida Tripodi, Roberta Fraschini, Monica Zocchi, Veronica Reghellin and Paola Coccetti

Supplementary Table S1. Yeast strain used in this study.

Strain	Genotype	Source
wt	*MATa ura3-52 trp1Δ63 his3Δ200 leu2Δ1 URA3::GFP-TUB1*	[1]
snf1Δ	*MATa ura3-52 trp1Δ63 his3Δ200 leu2Δ1 URA3::GFP-TUB1 snf1::HPH*	This study
Snf1-wt	*MATa leu2-3,112 ura3-1 trp1-1 his3-11,15 ade2-1 can1-100 snf1::HPH [pRS316-SNF1-HA]*	[2]
Snf1-T210A	*MATa leu2-3,112 ura3-1 trp1-1 his3-11,15 ade2-1 can1-100 snf1::HPH [pRS316-SNF1-T210A-HA]*	[2]
Snf1-as	*MATa leu2-3,112 ura3-1 trp1-1 his3-11,15 ade2-1 can1-100 snf1::HPH [pRS316-SNF1-I132G-HA]*	This study
Snf1-G53R	*MATa leu2-3,112 ura3-1 trp1-1 his3-11,15 ade2-1 can1-100 snf1::HPH [pRS316-SNF1-G53R-HA]*	[2]
Snf1-K84R	*MATa leu2-3,112 ura3-1 trp1-1 his3-11,15 ade2-1 can1-100 snf1::HPH [pRS316-SNF1-K84R-HA]*	[2]
Cdc3-HA	*MATa leu2-3,112 ura3-1 trp1-1 his3-11,15 ade2-1 can1-100 CDC3-HA::HIS3*	[3]
elm1Δ	*MATa leu2-3,112 ura3-1 trp1-1 his3-11,15 ade2-1 can1-100 elm1::NAT1*	[4]
cdc3-1	*MATa leu2-3,112 ura3-1 trp1-1 his3-11,15 ade2-1 can1-100 cdc3-1*	[5]
cdc12-6	*MATa leu2-3,112 ura3-1 trp1-1 his3-11,15 ade2-1 can1-100 cdc12-6*	[5]
bub2Δ	*MATa leu2-3,112 ura3-1 trp1-1 his3-11,15 ade2-1 can1-100 bub2::HIS3*	[6]
bub2Δsnf1Δ	*MATa leu2-3,112 ura3-1 trp1-1 his3-11,15 ade2-1 can1-100 bub2::HIS3 snf1::HPH*	This study
Elm1-eGFP	*MATa, ade2, trp1, can1, leu2-3, his3, ura3, elm1::eGFP*	[4]

Elm1-eGFP kar9Δ	*MAT a, ade2, trp1, can1, leu2-3, his3, ura3, elm1::eGFP, kar9::HIS3*	This study
kar9Δ	*MATa ura3-52 lys2-801 ade2-101 trp1Δ63 his3Δ200 leu2Δ1 kar9::klTRP1 ade2::ADE2-GFP-TUB1*	[7]
kar9Δsnf1Δ	*MATa ura3-52 lys2-801 ade2-101 trp1Δ63 his3Δ200 leu2Δ1 kar9::klTRP1 ade2::ADE2-GFP-TUB1 snf1::HPH*	This study
dyn1Δ	*MATa ura3-52 lys2-801 ade2-101 trp1Δ63 his3Δ200 leu2Δ1 URA3::GFP-TUB1 dyn1Δ::klTRP1*	[1]
dyn1Δsnf1Δ	*MATa ura3-52 lys2-801 ade2-101 trp1Δ63 his3Δ200 leu2Δ1 URA3::GFP-TUB1 dyn1Δ::klTRP1 snf1::HPH*	This study
dyn1Δsnf1Δ[Snf1-wt]	*MATa ura3-52 lys2-801 ade2-101 trp1Δ63 his3Δ200 leu2Δ1 URA3::GFP-TUB1 dyn1Δ::klTRP1 snf1::HPH [pRS313-SNF1-3HA]*	This study
kar9Δsnf1Δ[empty]	*MATa ura3-52 lys2-801 ade2-101 trp1Δ63 his3Δ200 leu2Δ1 kar9::klTRP1 ade2::ADE2-GFP-TUB1 snf1::HPH [pRS313]*	This study
kar9Δsnf1Δ[Snf1-wt]	*MATa ura3-52 lys2-801 ade2-101 trp1Δ63 his3Δ200 leu2Δ1 kar9::klTRP1 ade2::ADE2-GFP-TUB1 snf1::HPH [pRS313-SNF1-3HA]*	This study
kar9Δsnf1Δ[Snf1-G53R]	*MATa ura3-52 lys2-801 ade2-101 trp1Δ63 his3Δ200 leu2Δ1 kar9::klTRP1 ade2::ADE2-GFP-TUB1 snf1::HPH [pRS313-SNF1-G53R-3HA]*	This study
kar9Δ[pYX426]	*MATa ura3-52 lys2-801 ade2-101 trp1Δ63 his3Δ200 leu2Δ1 kar9::klTRP1 ade2::ADE2-GFP-TUB1 [pYX426]*	This study
kar9Δ[pSAK1-TAP]	*MATa ura3-52 lys2-801 ade2-101 trp1Δ63 his3Δ200 leu2Δ1 kar9::klTRP1 ade2::ADE2-GFP-TUB1 [pYX426-SAK1-TAP]*	This study
elm1Δ[pYX426]	*MATa leu2-3,112 ura3-1 trp1-1 his3-11,15 ade2-1 can1-100 elm1::NAT1 [pYX426]*	This study
elm1Δ[pSAK1-TAP]	*MATa leu2-3,112 ura3-1 trp1-1 his3-11,15 ade2-1 can1-100 elm1::NAT1 [pYX426-SAK1-TAP]*	This study
wt[pYX426]	*MATa leu2-3,112 ura3-1 trp1-1 his3-11,15 ade2-1 can1-100 [pYX426]*	This study
wt[pSAK1-TAP]	*MATa leu2-3,112 ura3-1 trp1-1 his3-11,15 ade2-1 can1-100 [pYX426-SAK1-TAP]*	This study

References

1. Pereira, G. & Schiebel, E. Kin4 kinase delays mitotic exit in response to spindle alignment defects. *Mol. Cell* **19,** 209–21 (2005).
2. Nicastro, R. *et al.* Snf1 Phosphorylates Adenylate Cyclase and Negatively Regulates Protein Kinase A-dependent Transcription in Saccharomyces cerevisiae. *J. Biol. Chem.* **290,** 24715–26 (2015).
3. Johnson, E. S. & Blobel, G. Cell cycle-regulated attachment of the ubiquitin-related protein SUMO to the

yeast septins. *J. Cell Biol.* **147,** 981–94 (1999).
4. Merlini, L. *et al.* Budding yeast dma proteins control septin dynamics and the spindle position checkpoint by promoting the recruitment of the Elm1 kinase to the bud neck. *PLoS Genet.* **8,** e1002670 (2012).
5. Cassani, C., Raspelli, E., Chiroli, E. & Fraschini, R. Vhs2 is a novel regulator of septin dynamics in budding yeast. *Cell Cycle* **13,** 1590–1601 (2014).
6. Fraschini, R., D'Ambrosio, C., Venturetti, M., Lucchini, G. & Piatti, S. Disappearance of the budding yeast Bub2–Bfa1 complex from the mother-bound spindle pole contributes to mitotic exit. *J. Cell Biol.* **172,** 335–346 (2006).
7. Caydasi, A. K. *et al.* Elm1 kinase activates the spindle position checkpoint kinase Kin4. *J. Cell Biol.* **190,** 975–89 (2010).

Supplementary Figures

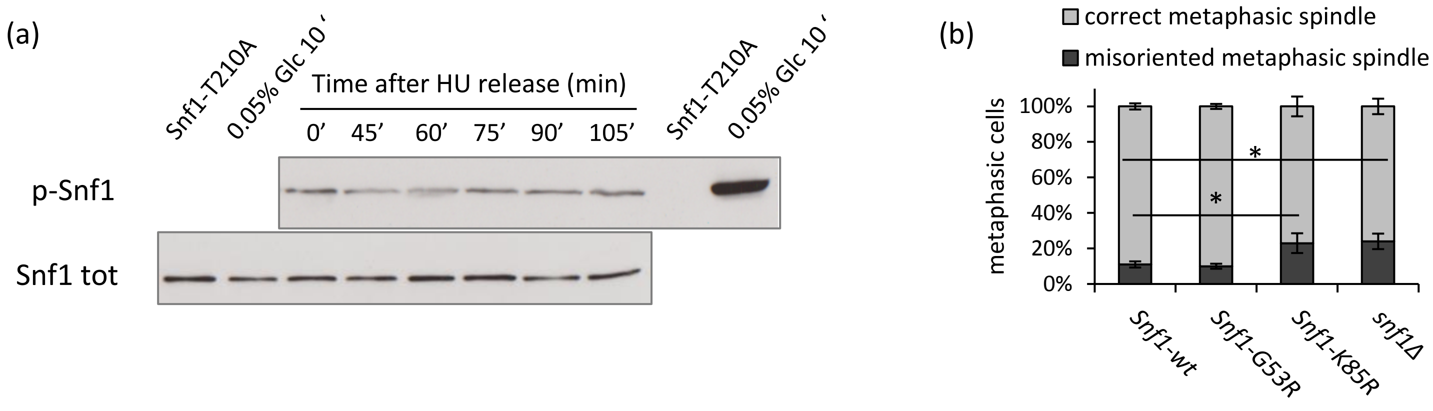

Supplementary Figure S1. (a) Non-cropped gels of Fig. 1A. A Snf1-HA expressing strain was grown in synthetic medium containing 2% glucose until exponential phase, then arrested and released from HU block as described in Materials and Methods. Samples were taken at different time points (0, 45, 60, 75, 90, 105 minutes) and analysed by western blot with anti-pT172-AMPK or anti-HA antibodies. Cells expressing Snf1-T210A-HA and Snf1-HA cells shifted to 0.05% glucose for 10 min were used as negative and positive control, respectively. (b) Quantification of misoriented metaphasic spindles in *Snf1-wt*, *Snf1-G53R*, *Snf1-K84R* and *snf1Δ* strains in exponential phase of growth.

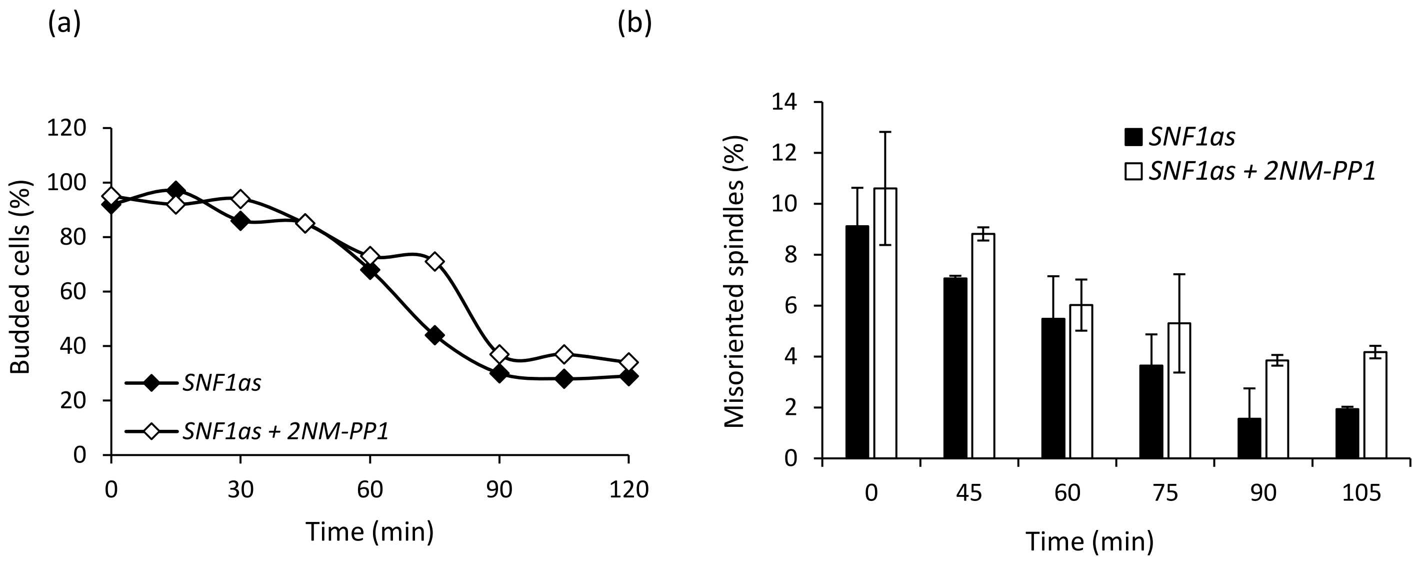

Supplementary Figure S2. (a-b) *Snf1-as* cells were grown in synthetic medium containing 5% glucose, synchronized in S phase by HU as described in Materials and Methods and released in fresh medium containing α-factor and either 25 µM 2NM-PP1 or 0.1% DMSO (solvent control). Samples were taken ate the indicated time points to assay (a) budding index, (b) spindle morphology.

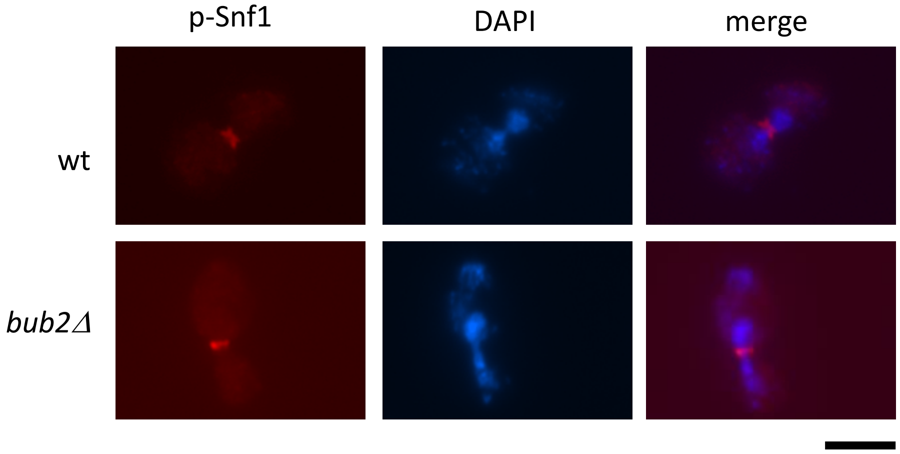

Supplementary Figure S3. Immunostaining for pT210-Snf1 using anti-pT172-AMPK antibody and DAPI staining of DNA in wt and *bub2Δ* cells. bar: 5 µm.

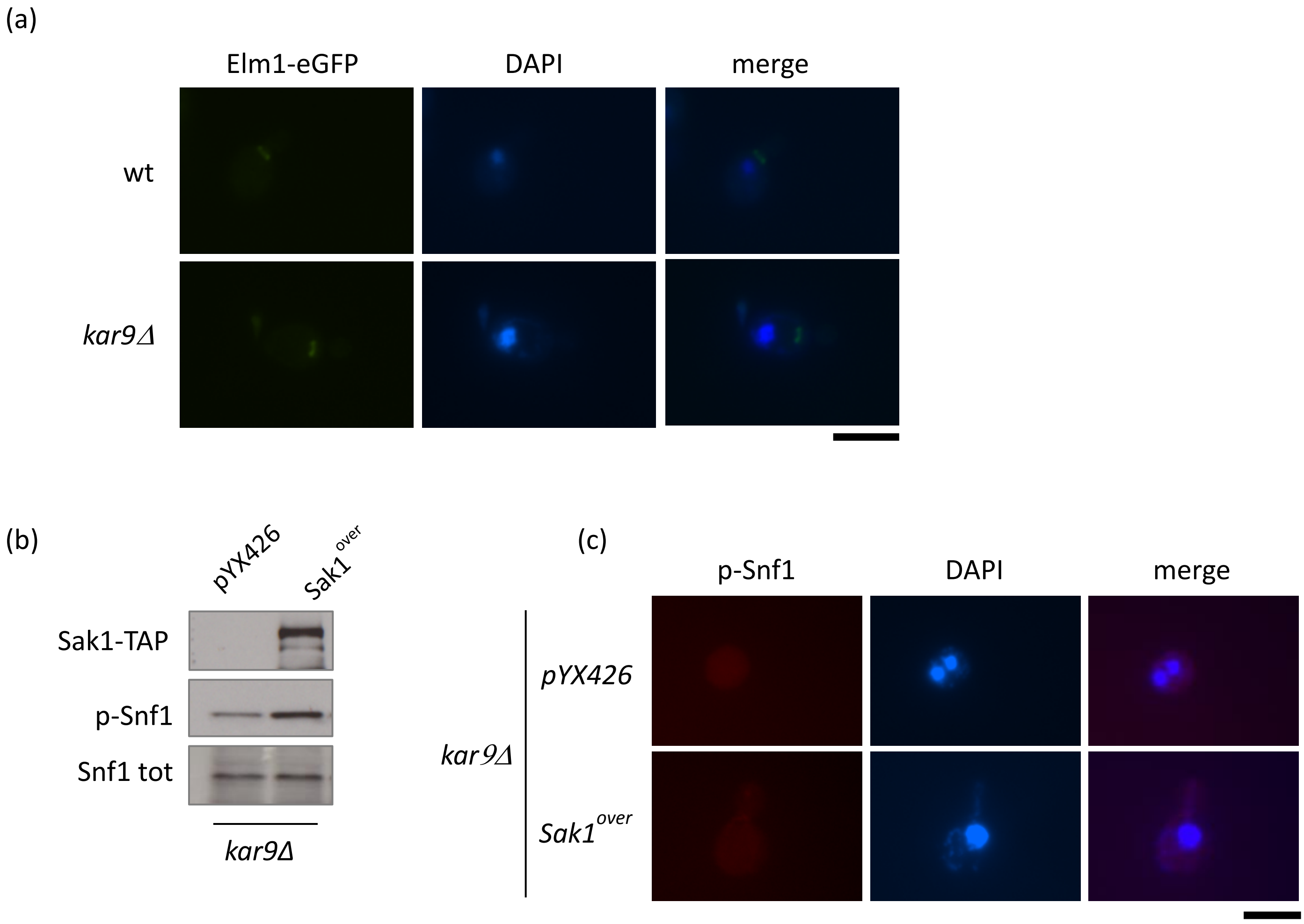

Supplementary Figure S4. (a) Elm1-eGFP visualization in wt and *kar9Δ* cells. DAPI was used for DNA staining. bar: 5 µm. (b-c) *kar9Δ[pYX426]* and *kar9Δ[pSAK1-TAP]* strains were grown in synthetic medium containing 2% glucose until exponential phase. Samples were taken to assay (b) SAK1-TAP, p-Snf1 and total Snf1 levels by western blot with anti-TAP, anti-pT172-AMPK and anti-His antibodies and (c) p-Snf1 localization by immunostaining using anti-pT172-AMPK antibody and DAPI staining of DNA. bar: 5 µm.

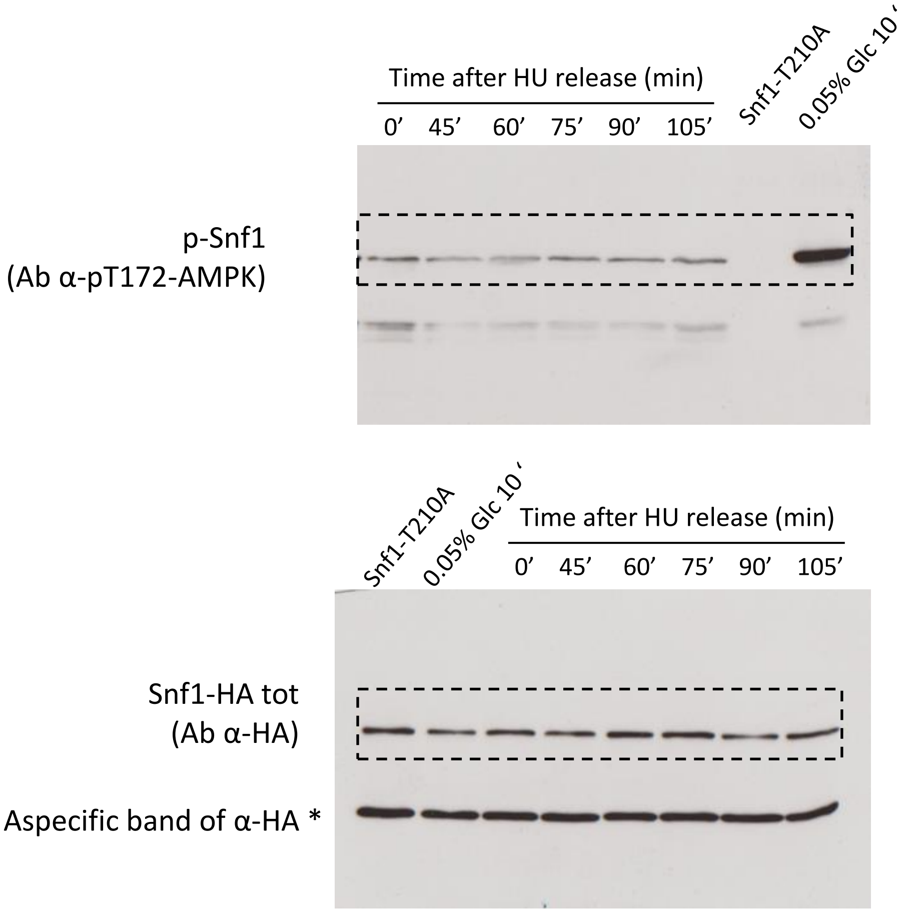

Supplementary Figure S5. Full blot images for Figure 1a with cropped regions marked with rectangles.

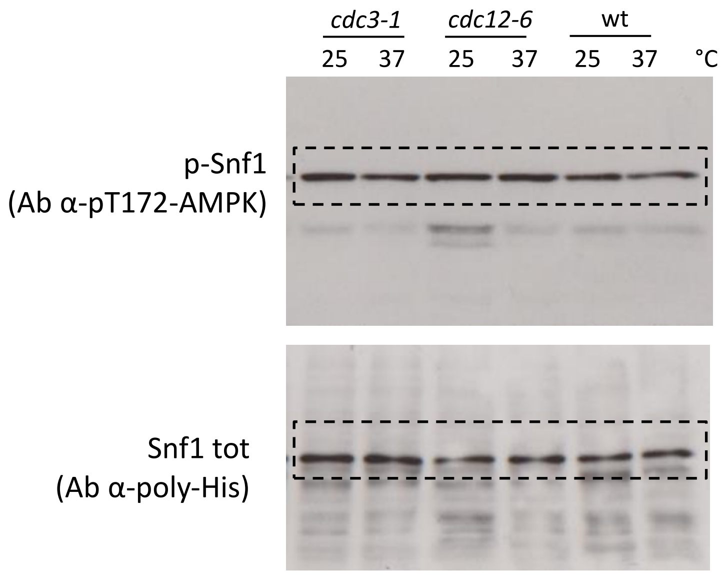

Supplementary Figure S6. Full blot images for Figure 2b with cropped regions marked with rectangles.

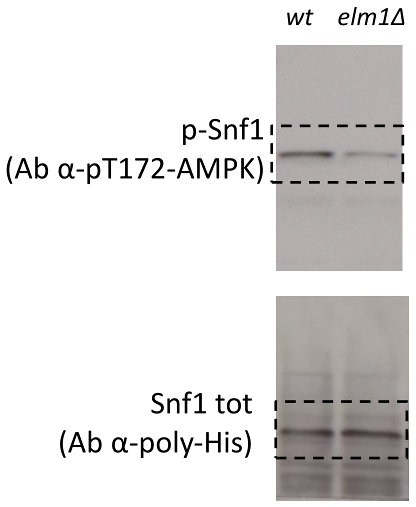

Supplementary Figure S7. Full blot images for Figure 2d with cropped regions marked with rectangles.

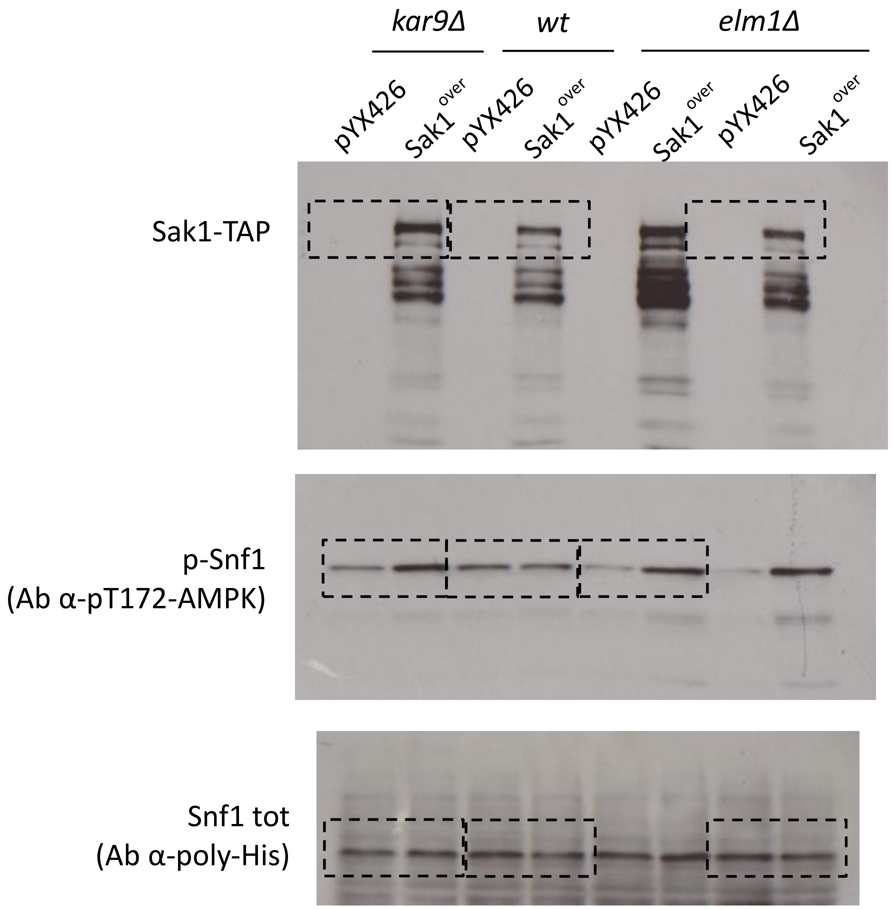

Supplementary Figure S8. Full blot images for Figure 2f and S4b with cropped regions marked with rectangles.

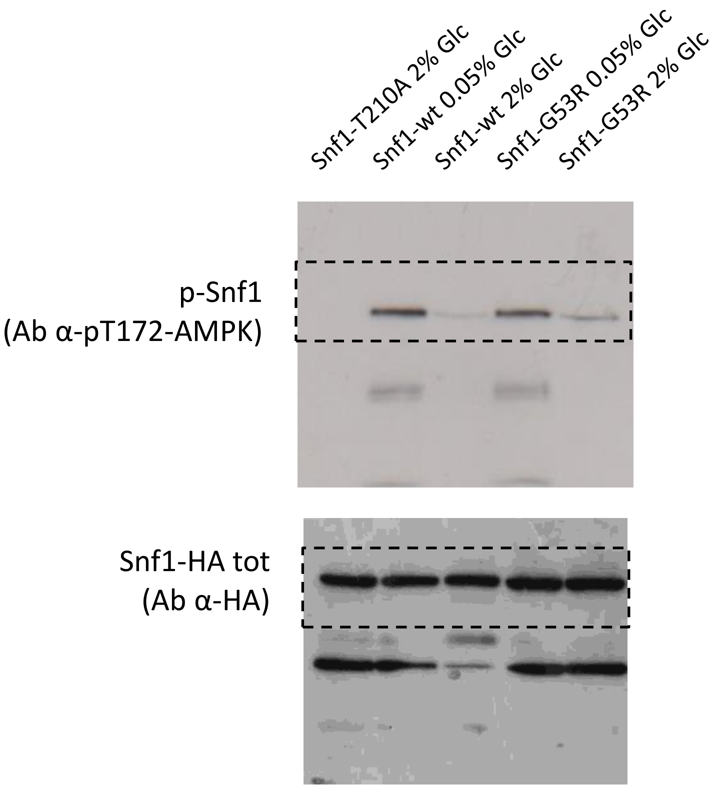

Supplementary Figure S9. Full blot images for Figure 4a with cropped regions marked with rectangles.

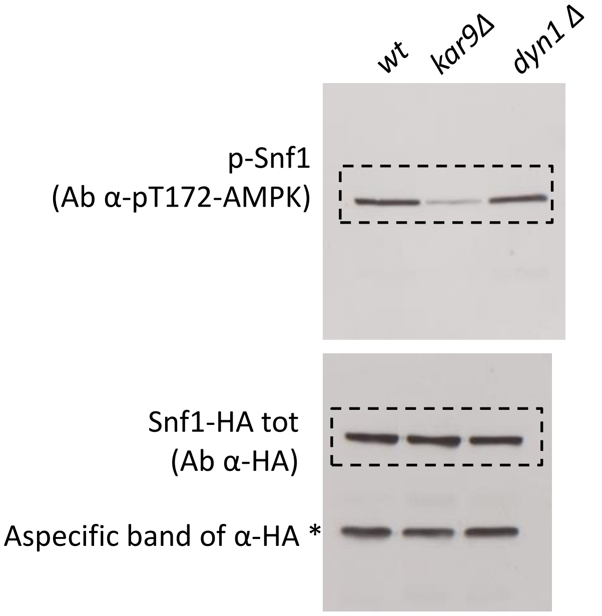

Supplementary Figure S10. Full blot images for Figure 7a with cropped regions marked with rectangles.
